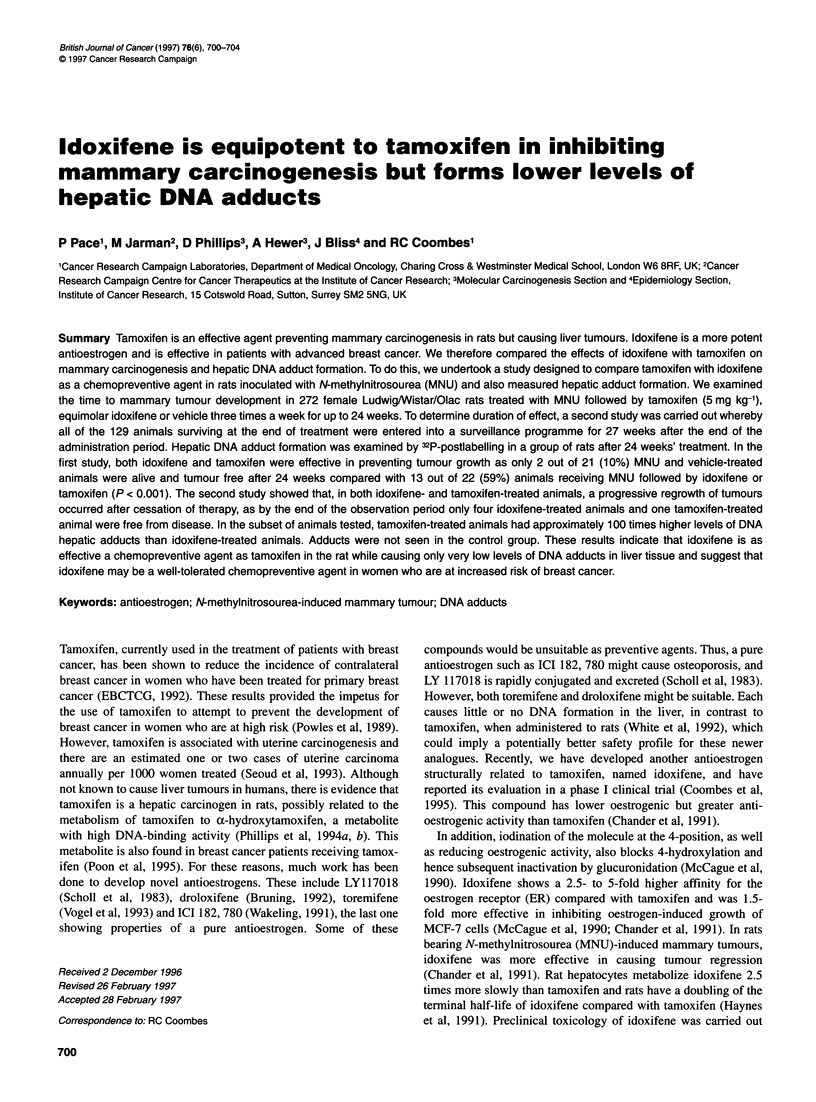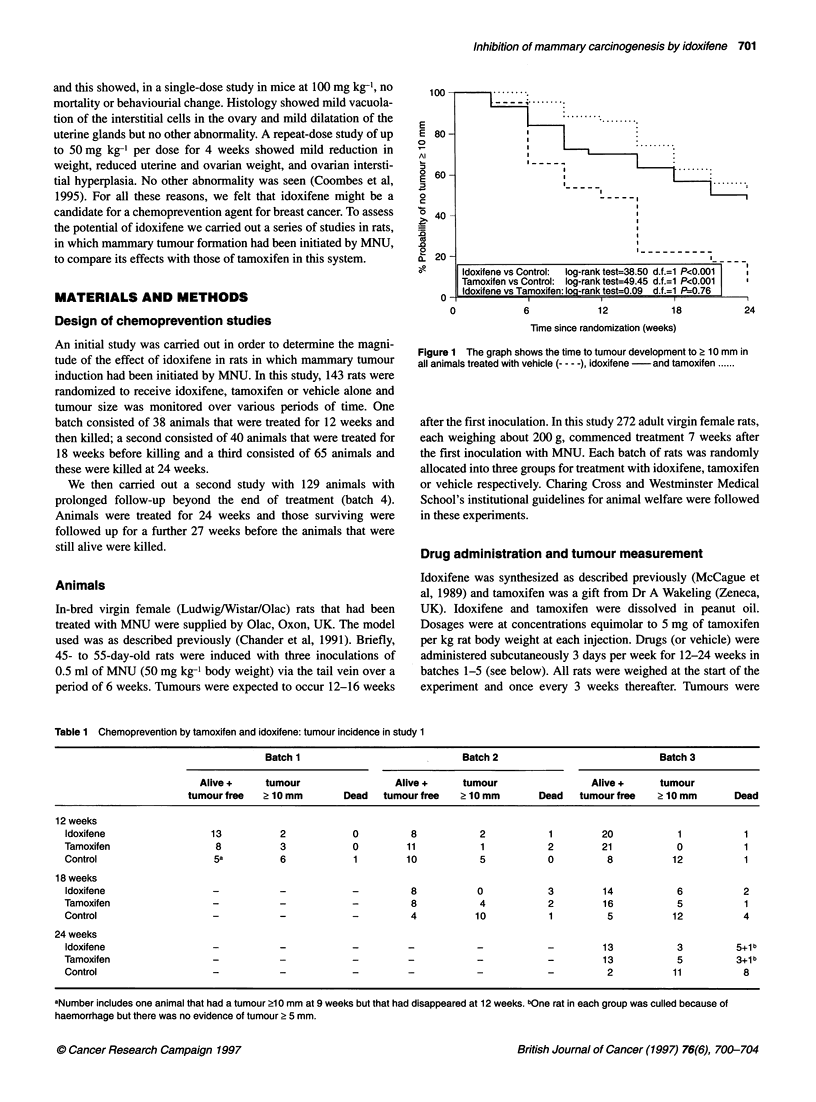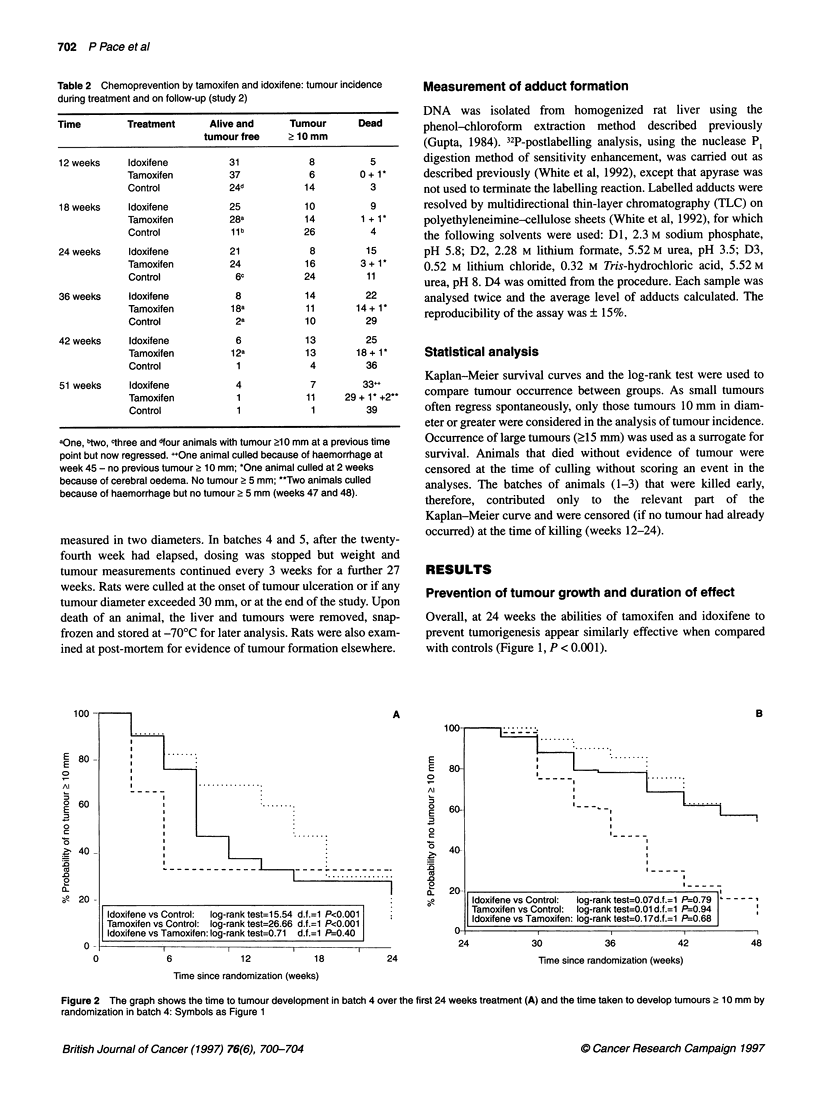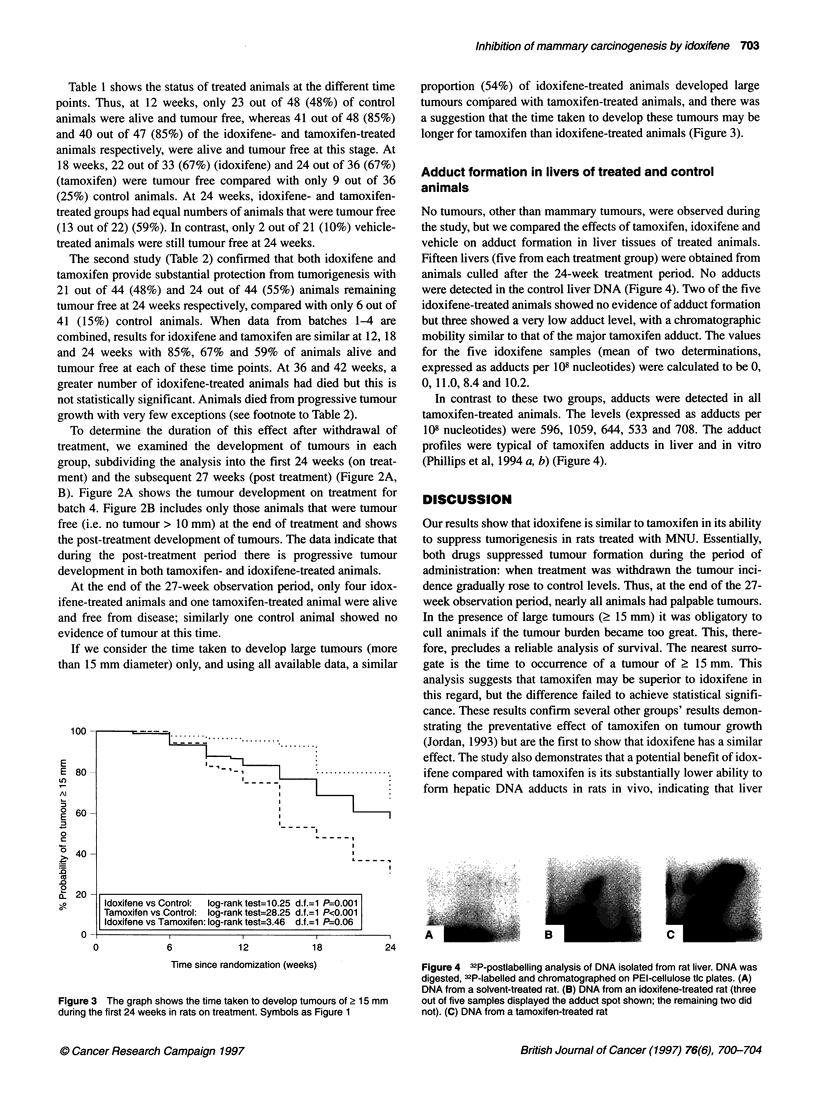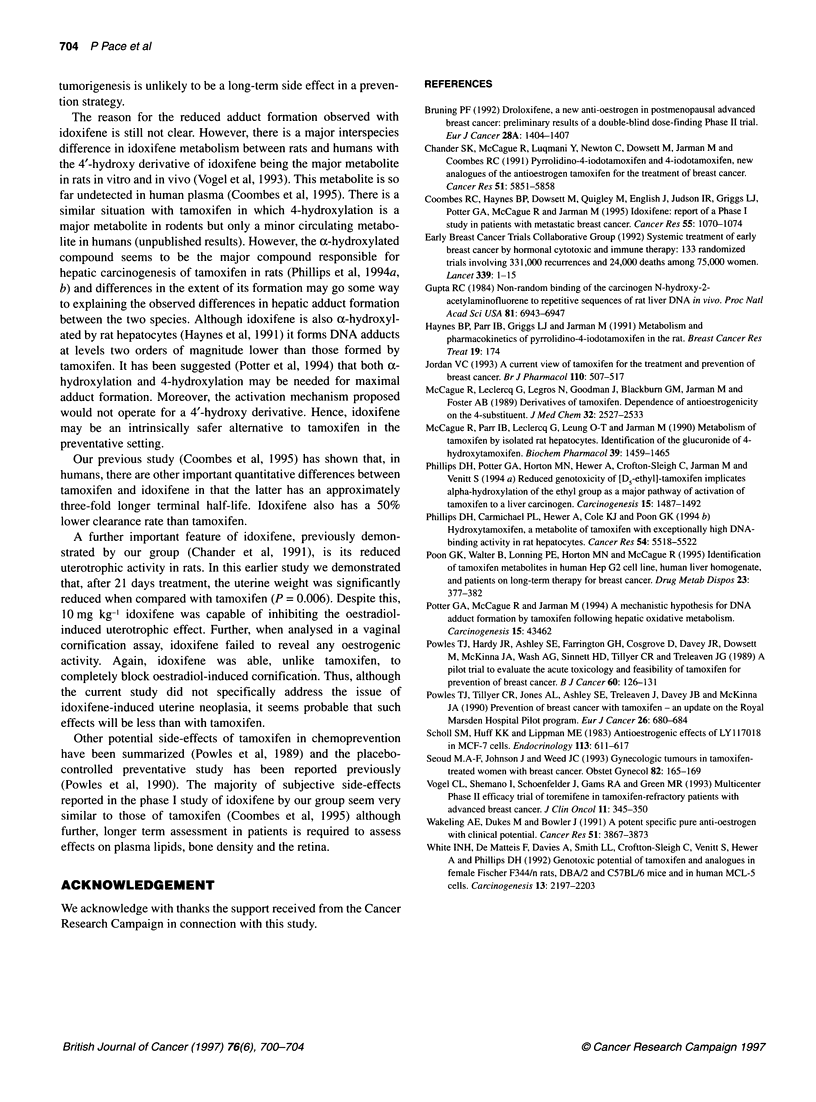# Idoxifene is equipotent to tamoxifen in inhibiting mammary carcinogenesis but forms lower levels of hepatic DNA adducts.

**DOI:** 10.1038/bjc.1997.449

**Published:** 1997

**Authors:** P. Pace, M. Jarman, D. Phillips, A. Hewer, J. Bliss, R. C. Coombes

**Affiliations:** Department of Medical Oncology, Charing Cross & Westminster Medical School, London, UK.

## Abstract

**Images:**


British Journal of Cancer (1997) 76(6), 700–704
© 1997 Cancer Research Campaign

### Idoxifene is equipotent to tamoxifen in inhibiting mammary carcinogenesis but forms lower levels of hepatic DNA adducts

**P Pace[1], M Jarman[2], D Phillips[3], A Hewer[3], J Bliss[4] and RC Coombes[1]**

[1]Cancer Research Campaign Laboratories, Department of Medical Oncology, Charing Cross & Westminster Medical School, London W6 8RF, UK; [2]Cancer Research Campaign Centre for Cancer Therapeutics at the Institute of Cancer Research; [3]Molecular Carcinogenesis Section and [4]Epidemiology Section, Institute of Cancer Research, 15 Cotswold Road, Sutton, Surrey SM2 5NG, UK

**Summary** Tamoxifen is an effective agent preventing mammary carcinogenesis in rats but causing liver tumours. Idoxifene is a more potent antioestrogen and is effective in patients with advanced breast cancer. We therefore compared the effects of idoxifene with tamoxifen on mammary carcinogenesis and hepatic DNA adduct formation. To do this, we undertook a study designed to compare tamoxifen with idoxifene as a chemopreventive agent in rats inoculated with *N*-methylnitrosourea (MNU) and also measured hepatic adduct formation. We examined the time to mammary tumour development in 272 female Ludwig/Wistar/Olac rats treated with MNU followed by tamoxifen (5 mg $kg^{-1}$), equimolar idoxifene or vehicle three times a week for up to 24 weeks. To determine duration of effect, a second study was carried out whereby all of the 129 animals surviving at the end of treatment were entered into a surveillance programme for 27 weeks after the end of the administration period. Hepatic DNA adduct formation was examined by $^{32}P$-postlabelling in a group of rats after 24 weeks' treatment. In the first study, both idoxifene and tamoxifen were effective in preventing tumour growth as only 2 out of 21 (10%) MNU and vehicle-treated animals were alive and tumour free after 24 weeks compared with 13 out of 22 (59%) animals receiving MNU followed by idoxifene or tamoxifen ($P < 0.001$). The second study showed that, in both idoxifene- and tamoxifen-treated animals, a progressive regrowth of tumours occurred after cessation of therapy, as by the end of the observation period only four idoxifene-treated animals and one tamoxifen-treated animal were free from disease. In the subset of animals tested, tamoxifen-treated animals had approximately 100 times higher levels of DNA hepatic adducts than idoxifene-treated animals. Adducts were not seen in the control group. These results indicate that idoxifene is as effective a chemopreventive agent as tamoxifen in the rat while causing only very low levels of DNA adducts in liver tissue and suggest that idoxifene may be a well-tolerated chemopreventive agent in women who are at increased risk of breast cancer.

**Keywords:** antioestrogen; *N*-methylnitrosourea-induced mammary tumour; DNA adducts

Tamoxifen, currently used in the treatment of patients with breast cancer, has been shown to reduce the incidence of contralateral breast cancer in women who have been treated for primary breast cancer (EBCTCG, 1992). These results provided the impetus for the use of tamoxifen to attempt to prevent the development of breast cancer in women who are at high risk (Powles et al, 1989). However, tamoxifen is associated with uterine carcinogenesis and there are an estimated one or two cases of uterine carcinoma annually per 1000 women treated (Seoud et al, 1993). Although not known to cause liver tumours in humans, there is evidence that tamoxifen is a hepatic carcinogen in rats, possibly related to the metabolism of tamoxifen to α-hydroxytamoxifen, a metabolite with high DNA-binding activity (Phillips et al, 1994*a*, *b*). This metabolite is also found in breast cancer patients receiving tamoxifen (Poon et al, 1995). For these reasons, much work has been done to develop novel antioestrogens. These include LY117018 (Scholl et al, 1983), droloxifene (Bruning, 1992), toremifene (Vogel et al, 1993) and ICI 182, 780 (Wakeling, 1991), the last one showing properties of a pure antioestrogen. Some of these compounds would be unsuitable as preventive agents. Thus, a pure antioestrogen such as ICI 182, 780 might cause osteoporosis, and LY 117018 is rapidly conjugated and excreted (Scholl et al, 1983). However, both toremifene and droloxifene might be suitable. Each causes little or no DNA formation in the liver, in contrast to tamoxifen, when administered to rats (White et al, 1992), which could imply a potentially better safety profile for these newer analogues. Recently, we have developed another antioestrogen structurally related to tamoxifen, named idoxifene, and have reported its evaluation in a phase I clinical trial (Coombes et al, 1995). This compound has lower oestrogenic but greater anti-oestrogenic activity than tamoxifen (Chander et al, 1991).

*Received 2 December 1996*
*Revised 26 February 1997*
*Accepted 28 February 1997*

*Correspondence to:* RC Coombes

In addition, iodination of the molecule at the 4-position, as well as reducing oestrogenic activity, also blocks 4-hydroxylation and hence subsequent inactivation by glucuronidation (McCague et al, 1990). Idoxifene shows a 2.5- to 5-fold higher affinity for the oestrogen receptor (ER) compared with tamoxifen and was 1.5-fold more effective in inhibiting oestrogen-induced growth of MCF-7 cells (McCague et al, 1990; Chander et al, 1991). In rats bearing *N*-methylnitrosourea (MNU)-induced mammary tumours, idoxifene was more effective in causing tumour regression (Chander et al, 1991). Rat hepatocytes metabolize idoxifene 2.5 times more slowly than tamoxifen and rats have a doubling of the terminal half-life of idoxifene compared with tamoxifen (Haynes et al, 1991). Preclinical toxicology of idoxifene was carried out and this showed, in a single-dose study in mice at 100 mg kg$^{-1}$, no mortality or behaviourial change. Histology showed mild vacuolation of the interstitial cells in the ovary and mild dilatation of the uterine glands but no other abnormality. A repeat-dose study of up to 50 mg kg$^{-1}$ per dose for 4 weeks showed mild reduction in weight, reduced uterine and ovarian weight, and ovarian interstitial hyperplasia. No other abnormality was seen (Coombes et al, 1995). For all these reasons, we felt that idoxifene might be a candidate for a chemoprevention agent for breast cancer. To assess the potential of idoxifene we carried out a series of studies in rats, in which mammary tumour formation had been initiated by MNU, to compare its effects with those of tamoxifen in this system.

### MATERIALS AND METHODS

### Design of chemoprevention studies

An initial study was carried out in order to determine the magnitude of the effect of idoxifene in rats in which mammary tumour induction had been initiated by MNU. In this study, 143 rats were randomized to receive idoxifene, tamoxifen or vehicle alone and tumour size was monitored over various periods of time. One batch consisted of 38 animals that were treated for 12 weeks and then killed; a second consisted of 40 animals that were treated for 18 weeks before killing and a third consisted of 65 animals and these were killed at 24 weeks.

We then carried out a second study with 129 animals with prolonged follow-up beyond the end of treatment (batch 4). Animals were treated for 24 weeks and those surviving were followed up for a further 27 weeks before the animals that were still alive were killed.

### Animals

In-bred virgin female (Ludwig/Wistar/Olac) rats that had been treated with MNU were supplied by Olac, Oxon, UK. The model used was as described previously (Chander et al, 1991). Briefly, 45- to 55-day-old rats were induced with three inoculations of 0.5 ml of MNU (50 mg kg$^{-1}$ body weight) via the tail vein over a period of 6 weeks. Tumours were expected to occur 12–16 weeks

**Figure 1** The graph shows the time to tumour development to ≥ 10 mm in all animals treated with vehicle (- - - -), idoxifene —— and tamoxifen ......

after the first inoculation. In this study 272 adult virgin female rats, each weighing about 200 g, commenced treatment 7 weeks after the first inoculation with MNU. Each batch of rats was randomly allocated into three groups for treatment with idoxifene, tamoxifen or vehicle respectively. Charing Cross and Westminster Medical School's institutional guidelines for animal welfare were followed in these experiments.

### Drug administration and tumour measurement

Idoxifene was synthesized as described previously (McCague et al, 1989) and tamoxifen was a gift from Dr A Wakeling (Zeneca, UK). Idoxifene and tamoxifen were dissolved in peanut oil. Dosages were at concentrations equimolar to 5 mg of tamoxifen per kg rat body weight at each injection. Drugs (or vehicle) were administered subcutaneously 3 days per week for 12–24 weeks in batches 1–5 (see below). All rats were weighed at the start of the experiment and once every 3 weeks thereafter. Tumours were

**Table 1** Chemoprevention by tamoxifen and idoxifene: tumour incidence in study 1

| | Batch 1 | | | Batch 2 | | | Batch 3 | | |
|---|---|---|---|---|---|---|---|---|---|
| | Alive + tumour free | tumour ≥ 10 mm | Dead | Alive + tumour free | tumour ≥ 10 mm | Dead | Alive + tumour free | tumour ≥ 10 mm | Dead |
| 12 weeks | | | | | | | | | |
| Idoxifene | 13 | 2 | 0 | 8 | 2 | 1 | 20 | 1 | 1 |
| Tamoxifen | 8 | 3 | 0 | 11 | 1 | 2 | 21 | 0 | 1 |
| Control | 5[a] | 6 | 1 | 10 | 5 | 0 | 8 | 12 | 1 |
| 18 weeks | | | | | | | | | |
| Idoxifene | – | – | – | 8 | 0 | 3 | 14 | 6 | 2 |
| Tamoxifen | – | – | – | 8 | 4 | 2 | 16 | 5 | 1 |
| Control | – | – | – | 4 | 10 | 1 | 5 | 12 | 4 |
| 24 weeks | | | | | | | | | |
| Idoxifene | – | – | – | – | – | – | 13 | 3 | 5+1[b] |
| Tamoxifen | – | – | – | – | – | – | 13 | 5 | 3+1[b] |
| Control | – | – | – | – | – | – | 2 | 11 | 8 |

[a]Number includes one animal that had a tumour ≥10 mm at 9 weeks but that had disappeared at 12 weeks. [b]One rat in each group was culled because of haemorrhage but there was no evidence of tumour ≥ 5 mm.

© Cancer Research Campaign 1997

**Table 2** Chemoprevention by tamoxifen and idoxifene: tumour incidence during treatment and on follow-up (study 2)

| Time | Treatment | Alive and tumour free | Tumour ≥ 10 mm | Dead |
|---|---|---|---|---|
| 12 weeks | Idoxifene | 31 | 8 | 5 |
| | Tamoxifen | 37 | 6 | 0 + 1* |
| | Control | 24[d] | 14 | 3 |
| 18 weeks | Idoxifene | 25 | 10 | 9 |
| | Tamoxifen | 28[a] | 14 | 1 + 1* |
| | Control | 11[b] | 26 | 4 |
| 24 weeks | Idoxifene | 21 | 8 | 15 |
| | Tamoxifen | 24 | 16 | 3 + 1* |
| | Control | 6[c] | 24 | 11 |
| 36 weeks | Idoxifene | 8 | 14 | 22 |
| | Tamoxifen | 18[a] | 11 | 14 + 1* |
| | Control | 2[a] | 10 | 29 |
| 42 weeks | Idoxifene | 6 | 13 | 25 |
| | Tamoxifen | 12[a] | 13 | 18 + 1* |
| | Control | 1 | 4 | 36 |
| 51 weeks | Idoxifene | 4 | 7 | 33++ |
| | Tamoxifen | 1 | 11 | 29 + 1* +2** |
| | Control | 1 | 1 | 39 |

[a]One, [b]two, [c]three and [d]four animals with tumour ≥10 mm at a previous time point but now regressed. ++One animal culled because of haemorrhage at week 45 – no previous tumour ≥ 10 mm; *One animal culled at 2 weeks because of cerebral oedema. No tumour ≥ 5 mm; **Two animals culled because of haemorrhage but no tumour ≥ 5 mm (weeks 47 and 48).

measured in two diameters. In batches 4 and 5, after the twenty-fourth week had elapsed, dosing was stopped but weight and tumour measurements continued every 3 weeks for a further 27 weeks. Rats were culled at the onset of tumour ulceration or if any tumour diameter exceeded 30 mm, or at the end of the study. Upon death of an animal, the liver and tumours were removed, snap-frozen and stored at –70°C for later analysis. Rats were also examined at post-mortem for evidence of tumour formation elsewhere.

### Measurement of adduct formation

DNA was isolated from homogenized rat liver using the phenol–chloroform extraction method described previously (Gupta, 1984). $^{32}$P-postlabelling analysis, using the nuclease $P_1$ digestion method of sensitivity enhancement, was carried out as described previously (White et al, 1992), except that apyrase was not used to terminate the labelling reaction. Labelled adducts were resolved by multidirectional thin-layer chromatography (TLC) on polyethyleneimine–cellulose sheets (White et al, 1992), for which the following solvents were used: D1, 2.3 M sodium phosphate, pH 5.8; D2, 2.28 M lithium formate, 5.52 M urea, pH 3.5; D3, 0.52 M lithium chloride, 0.32 M *Tris*-hydrochloric acid, 5.52 M urea, pH 8. D4 was omitted from the procedure. Each sample was analysed twice and the average level of adducts calculated. The reproducibility of the assay was ± 15%.

### Statistical analysis

Kaplan–Meier survival curves and the log-rank test were used to compare tumour occurrence between groups. As small tumours often regress spontaneously, only those tumours 10 mm in diameter or greater were considered in the analysis of tumour incidence. Occurrence of large tumours (≥15 mm) was used as a surrogate for survival. Animals that died without evidence of tumour were censored at the time of culling without scoring an event in the analyses. The batches of animals (1–3) that were killed early, therefore, contributed only to the relevant part of the Kaplan–Meier curve and were censored (if no tumour had already occurred) at the time of killing (weeks 12–24).

### RESULTS

### Prevention of tumour growth and duration of effect

Overall, at 24 weeks the abilities of tamoxifen and idoxifene to prevent tumorigenesis appear similarly effective when compared with controls (Figure 1, $P < 0.001$).

**Figure 2** The graph shows the time to tumour development in batch 4 over the first 24 weeks treatment (**A**) and the time taken to develop tumours ≥ 10 mm by randomization in batch 4: Symbols as Figure 1

© Cancer Research Campaign 1997

Table 1 shows the status of treated animals at the different time points. Thus, at 12 weeks, only 23 out of 48 (48%) of control animals were alive and tumour free, whereas 41 out of 48 (85%) and 40 out of 47 (85%) of the idoxifene- and tamoxifen-treated animals respectively, were alive and tumour free at this stage. At 18 weeks, 22 out of 33 (67%) (idoxifene) and 24 out of 36 (67%) (tamoxifen) were tumour free compared with only 9 out of 36 (25%) control animals. At 24 weeks, idoxifene- and tamoxifen-treated groups had equal numbers of animals that were tumour free (13 out of 22) (59%). In contrast, only 2 out of 21 (10%) vehicle-treated animals were still tumour free at 24 weeks.

The second study (Table 2) confirmed that both idoxifene and tamoxifen provide substantial protection from tumorigenesis with 21 out of 44 (48%) and 24 out of 44 (55%) animals remaining tumour free at 24 weeks respectively, compared with only 6 out of 41 (15%) control animals. When data from batches 1–4 are combined, results for idoxifene and tamoxifen are similar at 12, 18 and 24 weeks with 85%, 67% and 59% of animals alive and tumour free at each of these time points. At 36 and 42 weeks, a greater number of idoxifene-treated animals had died but this is not statistically significant. Animals died from progressive tumour growth with very few exceptions (see footnote to Table 2).

To determine the duration of this effect after withdrawal of treatment, we examined the development of tumours in each group, subdividing the analysis into the first 24 weeks (on treatment) and the subsequent 27 weeks (post treatment) (Figure 2A, B). Figure 2A shows the tumour development on treatment for batch 4. Figure 2B includes only those animals that were tumour free (i.e. no tumour > 10 mm) at the end of treatment and shows the post-treatment development of tumours. The data indicate that during the post-treatment period there is progressive tumour development in both tamoxifen- and idoxifene-treated animals.

At the end of the 27-week observation period, only four idoxifene-treated animals and one tamoxifen-treated animal were alive and free from disease; similarly one control animal showed no evidence of tumour at this time.

If we consider the time taken to develop large tumours (more than 15 mm diameter) only, and using all available data, a similar proportion (54%) of idoxifene-treated animals developed large tumours compared with tamoxifen-treated animals, and there was a suggestion that the time taken to develop these tumours may be longer for tamoxifen than idoxifene-treated animals (Figure 3).

**Figure 3** The graph shows the time taken to develop tumours of ≥ 15 mm during the first 24 weeks in rats on treatment. Symbols as Figure 1

### Adduct formation in livers of treated and control animals

No tumours, other than mammary tumours, were observed during the study, but we compared the effects of tamoxifen, idoxifene and vehicle on adduct formation in liver tissues of treated animals. Fifteen livers (five from each treatment group) were obtained from animals culled after the 24-week treatment period. No adducts were detected in the control liver DNA (Figure 4). Two of the five idoxifene-treated animals showed no evidence of adduct formation but three showed a very low adduct level, with a chromatographic mobility similar to that of the major tamoxifen adduct. The values for the five idoxifene samples (mean of two determinations, expressed as adducts per $10^8$ nucleotides) were calculated to be 0, 0, 11.0, 8.4 and 10.2.

In contrast to these two groups, adducts were detected in all tamoxifen-treated animals. The levels (expressed as adducts per $10^8$ nucleotides) were 596, 1059, 644, 533 and 708. The adduct profiles were typical of tamoxifen adducts in liver and in vitro (Phillips et al, 1994 *a, b*) (Figure 4).

### DISCUSSION

Our results show that idoxifene is similar to tamoxifen in its ability to suppress tumorigenesis in rats treated with MNU. Essentially, both drugs suppressed tumour formation during the period of administration: when treatment was withdrawn the tumour incidence gradually rose to control levels. Thus, at the end of the 27-week observation period, nearly all animals had palpable tumours. In the presence of large tumours (≥ 15 mm) it was obligatory to cull animals if the tumour burden became too great. This, therefore, precludes a reliable analysis of survival. The nearest surrogate is the time to occurrence of a tumour of ≥ 15 mm. This analysis suggests that tamoxifen may be superior to idoxifene in this regard, but the difference failed to achieve statistical significance. These results confirm several other groups' results demonstrating the preventative effect of tamoxifen on tumour growth (Jordan, 1993) but are the first to show that idoxifene has a similar effect. The study also demonstrates that a potential benefit of idoxifene compared with tamoxifen is its substantially lower ability to form hepatic DNA adducts in rats in vivo, indicating that liver

**Figure 4** $^{32}P$-postlabelling analysis of DNA isolated from rat liver. DNA was digested, $^{32}P$-labelled and chromatographed on PEI-cellulose tlc plates. (**A**) DNA from a solvent-treated rat. (**B**) DNA from an idoxifene-treated rat (three out of five samples displayed the adduct spot shown; the remaining two did not). (**C**) DNA from a tamoxifen-treated rat

© Cancer Research Campaign 1997

tumorigenesis is unlikely to be a long-term side effect in a prevention strategy.

The reason for the reduced adduct formation observed with idoxifene is still not clear. However, there is a major interspecies difference in idoxifene metabolism between rats and humans with the 4′-hydroxy derivative of idoxifene being the major metabolite in rats in vitro and in vivo (Vogel et al, 1993). This metabolite is so far undetected in human plasma (Coombes et al, 1995). There is a similar situation with tamoxifen in which 4-hydroxylation is a major metabolite in rodents but only a minor circulating metabolite in humans (unpublished results). However, the α-hydroxylated compound seems to be the major compound responsible for hepatic carcinogenesis of tamoxifen in rats (Phillips et al, 1994*a*, *b*) and differences in the extent of its formation may go some way to explaining the observed differences in hepatic adduct formation between the two species. Although idoxifene is also α-hydroxylated by rat hepatocytes (Haynes et al, 1991) it forms DNA adducts at levels two orders of magnitude lower than those formed by tamoxifen. It has been suggested (Potter et al, 1994) that both α-hydroxylation and 4-hydroxylation may be needed for maximal adduct formation. Moreover, the activation mechanism proposed would not operate for a 4′-hydroxy derivative. Hence, idoxifene may be an intrinsically safer alternative to tamoxifen in the preventative setting.

Our previous study (Coombes et al, 1995) has shown that, in humans, there are other important quantitative differences between tamoxifen and idoxifene in that the latter has an approximately three-fold longer terminal half-life. Idoxifene also has a 50% lower clearance rate than tamoxifen.

A further important feature of idoxifene, previously demonstrated by our group (Chander et al, 1991), is its reduced uterotrophic activity in rats. In this earlier study we demonstrated that, after 21 days treatment, the uterine weight was significantly reduced when compared with tamoxifen ($P = 0.006$). Despite this, 10 mg $kg^{-1}$ idoxifene was capable of inhibiting the oestradiol-induced uterotrophic effect. Further, when analysed in a vaginal cornification assay, idoxifene failed to reveal any oestrogenic activity. Again, idoxifene was able, unlike tamoxifen, to completely block oestradiol-induced cornification. Thus, although the current study did not specifically address the issue of idoxifene-induced uterine neoplasia, it seems probable that such effects will be less than with tamoxifen.

Other potential side-effects of tamoxifen in chemoprevention have been summarized (Powles et al, 1989) and the placebo-controlled preventative study has been reported previously (Powles et al, 1990). The majority of subjective side-effects reported in the phase I study of idoxifene by our group seem very similar to those of tamoxifen (Coombes et al, 1995) although further, longer term assessment in patients is required to assess effects on plasma lipids, bone density and the retina.

### ACKNOWLEDGEMENT

We acknowledge with thanks the support received from the Cancer Research Campaign in connection with this study.

### REFERENCES

Bruning PF (1992) Droloxifene, a new anti-oestrogen in postmenopausal advanced breast cancer: preliminary results of a double-blind dose-finding Phase II trial. *Eur J Cancer* **28A**: 1404–1407

Chander SK, McCague R, Luqmani Y, Newton C, Dowsett M, Jarman M and Coombes RC (1991) Pyrrolidino-4-iodotamoxifen and 4-iodotamoxifen, new analogues of the antioestrogen tamoxifen for the treatment of breast cancer. *Cancer Res* **51**: 5851–5858

Coombes RC, Haynes BP, Dowsett M, Quigley M, English J, Judson IR, Griggs LJ, Potter GA, McCague R and Jarman M (1995) Idoxifene: report of a Phase I study in patients with metastatic breast cancer. *Cancer Res* **55**: 1070–1074

Early Breast Cancer Trials Collaborative Group (1992) Systemic treatment of early breast cancer by hormonal cytotoxic and immune therapy: 133 randomized trials involving 331,000 recurrences and 24,000 deaths among 75,000 women. *Lancet* **339**: 1–15

Gupta RC (1984) Non-random binding of the carcinogen N-hydroxy-2-acetylaminofluorene to repetitive sequences of rat liver DNA *in vivo. Proc Natl Acad Sci USA* **81**: 6943–6947

Haynes BP, Parr IB, Griggs LJ and Jarman M (1991) Metabolism and pharmacokinetics of pyrrolidino-4-iodotamoxifen in the rat. *Breast Cancer Res Treat* **19**: 174

Jordan VC (1993) A current view of tamoxifen for the treatment and prevention of breast cancer. *Br J Pharmacol* **110**: 507–517

McCague R, Leclercq G, Legros N, Goodman J, Blackburn GM, Jarman M and Foster AB (1989) Derivatives of tamoxifen. Dependence of antioestrogenicity on the 4-substituent. *J Med Chem* **32**: 2527–2533

McCague R, Parr IB, Leclercq G, Leung O-T and Jarman M (1990) Metabolism of tamoxifen by isolated rat hepatocytes. Identification of the glucuronide of 4-hydroxytamoxifen. *Biochem Pharmacol* **39**: 1459–1465

Phillips DH, Potter GA, Horton MN, Hewer A, Crofton-Sleigh C, Jarman M and Venitt S (1994 *a*) Reduced genotoxicity of [$D_5$-ethyl]-tamoxifen implicates alpha-hydroxylation of the ethyl group as a major pathway of activation of tamoxifen to a liver carcinogen. *Carcinogenesis* **15**: 1487–1492

Phillips DH, Carmichael PL, Hewer A, Cole KJ and Poon GK (1994 *b*) Hydroxytamoxifen, a metabolite of tamoxifen with exceptionally high DNA-binding activity in rat hepatocytes. *Cancer Res* **54**: 5518–5522

Poon GK, Walter B, Lonning PE, Horton MN and McCague R (1995) Identification of tamoxifen metabolites in human Hep G2 cell line, human liver homogenate, and patients on long-term therapy for breast cancer. *Drug Metab Dispos* **23**: 377–382

Potter GA, McCague R and Jarman M (1994) A mechanistic hypothesis for DNA adduct formation by tamoxifen following hepatic oxidative metabolism. *Carcinogenesis* **15**: 43462

Powles TJ, Hardy JR, Ashley SE, Farrington GH, Cosgrove D, Davey JR, Dowsett M, McKinna JA, Wash AG, Sinnett HD, Tillyer CR and Treleaven JG (1989) A pilot trial to evaluate the acute toxicology and feasibility of tamoxifen for prevention of breast cancer. *B J Cancer* **60**: 126–131

Powles TJ, Tillyer CR, Jones AL, Ashley SE, Treleaven J, Davey JB and McKinna JA (1990) Prevention of breast cancer with tamoxifen – an update on the Royal Marsden Hospital Pilot program. *Eur J Cancer* **26**: 680–684

Scholl SM, Huff KK and Lippman ME (1983) Antioestrogenic effects of LY117018 in MCF-7 cells. *Endocrinology* **113**: 611–617

Seoud M.A-F, Johnson J and Weed JC (1993) Gynecologic tumours in tamoxifen-treated women with breast cancer. Obstet Gynecol **82**: 165–169

Vogel CL, Shemano I, Schoenfelder J, Gams RA and Green MR (1993) Multicenter Phase II efficacy trial of toremifene in tamoxifen-refractory patients with advanced breast cancer. *J Clin Oncol* **11**: 345–350

Wakeling AE, Dukes M and Bowler J (1991) A potent specific pure anti-oestrogen with clinical potential. *Cancer Res* **51**: 3867–3873

White INH, De Matteis F, Davies A, Smith LL, Croftton-Sleigh C, Venitt S, Hewer A and Phillips DH (1992) Genotoxic potential of tamoxifen and analogues in female Fischer F344/n rats, DBA/2 and C57BL/6 mice and in human MCL-5 cells. *Carcinogenesis* **13**: 2197–2203

© Cancer Research Campaign 1997